# A Review of Virtual Medical Student Rotations During the COVID-19 Pandemic: Their Role, Advantages, Disadvantages, and Future Prospects

**DOI:** 10.7759/cureus.24280

**Published:** 2022-04-19

**Authors:** Travis Satnarine, Che Marie Lee Kin

**Affiliations:** 1 Neonatal Intensive Care Unit, Port of Spain General Hospital, Port of Spain, TTO

**Keywords:** virtual academics, virtual learning environment, virtual learning, virtual teaching, virtual clinical rotations, virtual observership, virtual clerkship, virtual rotation, tele rotation

## Abstract

The COVID-19 pandemic resulted in the pause of medical clinical rotations. As a result, virtual rotations were implemented. These are a form of remote learning that seeks to mimic the clinical learning environment that students were already accustomed to.

This article seeks to review the published literature to explore which specialties adapted this format, what are the advantages and disadvantages observed, determine what were the responsibilities and involvements of students participating in these rotations, how well these rotations substituted for in-person rotations, and to evaluate if there is a continued role for them after, outside of COVID-19.

Virtual rotations have been developed in almost every specialty. These rotations have been developed from small centers to large universities, and are widespread throughout the United States, and in other countries as well. These rotations are targeted toward medical students, medical residents, and physician assistants and range in length from one to four weeks. Responsibilities and scope of interaction varied according to rotation; some rotations allowed patient interaction, and observation of procedures and surgeries, whereas some were purely didactic. A mixture of inpatient and outpatient involvements was seen.

Advantages included saving money and time, more flexibility, increased diversity, and participation of international medical graduates. Virtual rotations participants have been invited for interviews at the participating institution's residency programs and have matched there. Disadvantages included lack of assessment of practical skills, inability to receive credit, and inability to obtain a letter of recommendation.

Virtual rotations have proven to be a good substitute for in-person rotations, with most medical students seeing a need for the rotations in the future. Due to widespread development and acceptance of these rotations, it is likely that these rotations will continue.

## Introduction and background

In early 2020, the World Health Organization (WHO) announced concerns of a new pneumonia that appeared to have started in Wuhan, China. This was attributed to a new strain of coronavirus which would later become known as “COVID-19” (Coronavirus Disease-2019). The WHO declared the threat a global health emergency, and this led to the restriction of travel and decreased movement of people. However, as the human-to-human transmission continued, in March 2020, the crisis escalated and became classified as a pandemic [1]. With global calls for quarantines, and stay-at-home orders being implemented in the United States, to curb the spread of the virus, everyday life was disrupted. As daily interactions shifted to an online format, the Centers for Medicare & Medicaid Services (CMS) announced the relaxation of measures to cover virtual interactions between doctors and patients [1].

The world of medical education was similarly affected. The Association of American Medical Colleges (AAMC) called for an immediate cessation of medical students participating in patient care and clinical rotations in an effort to protect the students as well as the patients, to conserve personal protective equipment (PPE), to gather more information on the novel virus, and to formulate strategies for medical learning in the midst of the pandemic [2]. This pause in clinical clerkships was a sentiment that was echoed at medical schools around the world. The pandemic encouraged the further modernization of medical education leading to advances such as the introduction of virtual rotations, which are sometimes known as “tele-rotations”. These are a form of remote learning that seeks to emulate the clinical environment that students were accustomed to during their rotations [3].

This article seeks to review the published literature to explore which specialties adapted this format, what are the advantages and disadvantages observed, and determine what were the responsibilities and involvements of students participating in these rotations. It is hoped that by answering these questions, we can evaluate how well these rotations have filled the gap that was left by the COVID-19 pandemic, how well these rotations substituted for in-person rotations, and evaluate if there is a continued role for them after, outside of COVID-19.

## Review

Methodology

Search Strategy

The databases PubMed, MEDLINE, and PubMed Central (PMC) were utilized to source relevant literature for this review. They were searched using the predetermined keywords “tele rotation” and “virtual rotation”. Medical Subject Headings (MeSH) of these terms yielded no results.

The search term “tele rotation” yielded 36 results. These articles were screened initially by title screen. Five articles were screened based on title, but only three of these abstracts were found to be relevant. Once the abstracts were relevant, the studies were included. A total of three studies were included from this search. All three of these full-text articles were available. Then, the search term “virtual rotation” yielded 2,242 results. Automatic filters (5 years, English, Humans) reduced the number of studies to 597 articles. A total of 39 articles were screened based on their title. Of these, 20 articles were found to be relevant based on the abstract screen and were included in this study. Of the 20 studies, 18 full-text articles were available. There were no duplicate articles among the included studies. The articles did not undergo quality screening. In total, 23 studies were included.

Eligibility

Inclusion criteria included articles that were already published, peer-reviewed, in the English language, or if an English translation was available, and related to the specialties of human medicine. Irrelevant articles and grey literature were not included. Articles obtained were published between July 2020 and March 2022.

Results

Virtual rotations have been developed in almost every specialty, including but not limited to emergency medicine [4,5], geriatric psychiatry [6], intensive care [3], internal medicine [7], obstetrics and gynecology [8], ophthalmology [9], orthopedics [10,11], otolaryngology [12,13], pathology [14,15], pediatrics [16], physical medicine and rehabilitation [17], plastic surgery [18], radiation oncology [19,20], radiology [21], telehealth [22], urology [23,24], and vascular surgery [25].

These rotations cater to a clientele of medical students [3-5,8-25], resident physicians [6,23], and physician assistants [7]. They ranged in length from one week [24], two weeks [13,16-18,20], three weeks [8,15], four weeks [3,5,9,14,21,22], or were not specified. Responsibilities and scope of interaction varied according to rotation and are explored in Table 1. Some rotations allowed direct patient interaction, and some were purely didactic in nature. The rotations utilized a wide variety of virtual platforms, of which Zoom (Zoom Video Communications, Inc, San Jose, California, USA) was the most popular - refer to Table 2.

**Table 1:** Virtual rotations listed by specialty (in alphabetical order) * Duration of rotation was eight sessions of two hours each. ** There were sessions, each 1.5-2 hours long, every 2 weeks.

Specialty	Author, year	Institute	Location	Weeks	Scope of Educational Activities
Emergency Medicine	Redinger and Greene, 2021 [5]	Western Michigan University Homer Stryker M.D. School of Medicine	Kalamazoo, Michigan	4	Self-directed learning and case discussions of simulated patients.
	Villa et al., 2021 [4]	The University of California, Los Angeles	Los Angeles, California	2	Didactic teaching sessions, professional development, case discussions, introduction to their residency program.
Geriatric Psychiatry	Collier, 2020 [6]	McLean Hospital Division of Geriatric Psychiatry	Belmont, Massachusetts	-	Outpatient setting with patient interaction, didactic teaching sessions, presentations.
Intensive Care Unit	Ho et al., 2021 [3]	The Perelman School of Medicine at the University of Pennsylvania	Philadelphia, Pennsylvania	4	Didactic teaching sessions, formal assessments, presentations, and evidence-based medicine discussions.
Internal Medicine	Saltzman et al., 2021 [7]	The University of Chicago	Chicago, Illinois	-	Didactic teaching sessions, patient interaction, case discussions.
Obstetrics and Gynecology	Armon et al., 2021 [8]	The Hebrew University in Jerusalem	Jerusalem, Israel	3	Didactic teaching sessions, problem-based learning sessions, and case discussions.
Ophthalmology	DeVaro et al., 2020 [9]	The Emory University School of Medicine	Atlanta, Georgia	4	Self-directed learning, student presentations, case discussions, chart review activities with access to the Electronic Medical Record (EMR). Patient engagement was offered whereby the student was able to interact with a patient and remotely perform history taking and focused physical examination.
Orthopedics	Mason and Aruma, 2022 [11]	Penn State Health Milton S. Hershey Medical Center	Hershey, Pennsylvania	*	Presentations and case discussions.
	Yellin et al., 2022 [10]	Harvard Combined Orthopaedic Residency Program (Massachusetts General Hospital, Brigham and Women's Hospital, Boston Children's Hospital, Beth Israel Deaconess Medical Center).	Boston Massachusetts	3	Series of didactic teaching sessions, led by faculty members, ability to introduce the rotating students to the faculty and vice versa.
Otolaryngology	Chao et al., 2021 [12]	The University of Pennsylvania	Philadelphia, Pennsylvania	-	Out-patient settings with patient interaction with history-taking and case presentations, observation of surgical procedures in the operating theatre with live streaming, didactic teaching sessions and discussions.
	Shah et al., 2022 [13]	Yale University School of Medicine	New Haven, Connecticut	2	Observation of surgical procedures.
Pathology	Koch et al., 2022 [14]	University of Washington Medical Center	Seattle, Washington	4	Didactic teaching sessions, lectures, autopsy cases, and viewing virtual slide trays.
	White et al., 2021 [15]	Johns Hopkins University School of Medicine	Baltimore, Maryland	3	Comprehensive review of basics, lectures, case discussions, and presentations.
Pediatrics	Peter-Kern et al., 2020 [16]	Kinderpoliklinik Universitätsklinikum Würzburg Kinderklinik	Würzburg, Germany	2	Interact with inpatients and outpatients, history-taking, visual diagnosis, case discussions.
Physical Medicine and Rehabilitation	Huang et al., 2021 [17]	University of Miami	Miami, Florida	2	Participation in inpatient rounds, electrodiagnostic medicine, musculoskeletal medicine and ultrasound clinics, and presentations
Plastic Surgery	Song and Haley, 2020 [18]	The University of California, San Diego	La Jolla, California	2	Case discussions, mentor meetings, and social activities
Radiation Oncology	Janopaul-Naylor et al., 2021 [20]	The Winship Cancer Institute at Emory University	Atlanta, Georgia	2	Patient engagement, observation of procedures, presentations, and discussions
	Kahn et al., 2021 [19]	Radiation Oncology Virtual Education Rotation (ROVER)	Multiple states, United States	**	Case-based discussions.
Radiology	Creagh et al., 2021 [21]	Aventura Hospital and Medical Center	Aventura, Florida	4	Pre-recorded lectures, reading material, case discussions.
Telehealth	Weber et al., 2021 [22]	The Rutgers Robert Wood Johnson Medical School	New Brunswick, New Jersey	4	Understanding the applications of telehealth and learning its technologies, assessing patient clinical status, and developing a plan of action and care for patients.
Urology	Manalo et al., 2020 [24]	Emory University School of Medicine	Atlanta, Georgia	1	Lectures and discussion of cases
	Margolin et al., 2021 [23]	Columbia University Irving Medical Center	New York, New York	4	Conferences, didactic teaching sessions, virtual encounters with patients, and interactive online activities. The clinical engagements allowed virtual observation of operating room procedures, outpatient history-taking, and observation of outpatient procedures
Vascular Surgery	Patel et al., 2021 [25]	The Louisiana State University Health Sciences Center	New Orleans, Louisiana	-	Interactive teachings, conferences, surgical videos, and sessions learning to sutures and tie knots

**Table 2:** Platforms used during virtual rotations

Virtual platform	Company	Ref.
Microsoft Teams	Microsoft Corporation, Redmond, Washington, USA	[5,14]
Zoom	Zoom Video Communications, Inc, San Jose, California, USA	[4,6-10,14,15,17,19,20,23-25]
eCareManager	Philips Healthcare, Amsterdam, The Netherlands	[3]
FaceTime	Apple Inc., Cupertino, California, USA	[7]
BlueJeans	BlueJeans, Verizon Enterprise Solutions LLC, Mountainview, California, USA	[12]
Doximity	Video Dialer Beta, Doximity Inc., San Francisco, California, USA	[12,22]
Skype for Business	Microsoft Corporation, Redmond, Washington, USA	[16]
Cisco	Cisco Systems, San Jose, California, USA	[23]

Discussion

The format of virtual rotations seeks to mimic those of in-person rotations [14]. A mixture of inpatient and outpatient involvements was seen. Some rotations encouraged learning through interacting with patients, similar to the format of traditional in-person rotations, while some limited patient engagement and instead opted for a hybrid approach or an entirely didactic and simulated environment. This format was adopted not only throughout the United States but also in some countries such as Israel [8] and Germany [16]. The rotations were prevalent throughout many medical schools and universities in the United States as seen in Table 1. Similar to the safety practices that were necessary for in-person rotations, institutions utilized various combinations of necessary prerequisites, including but not limited to: the Health Insurance Portability and Accountability Act (HIPAA) training, confidentiality and privacy agreements, participant background checks, and malpractice insurance coverage [10,14,25].

Advantages and Benefits

There were many notable advantages and benefits of virtual rotations, for medical students including less cost, due to saving money that would have been spent on rent and travel, less time-consuming, with some rotations requiring as little as 2 hours per day commitment, more one-on-one time with attendings, as compared to in-person rotations which encouraged more one-on-one time with residents, students were able to obtain a sense of the culture of the program, more students were allowed to participate, than in previous years with in-person rotations only, the program’s schedule was more flexible, and hence this allowed students to also attend other required rotations. Other advantages included increased diversity and the increased ability of international medical graduates to attend [3,4,12,17,26].

Students were able to participate in clinical management, including suggesting commonly used medications, and ventilator setting adjustments, while being kept safe from the virus, and without utilizing PPE [3]. Students cited beneficial aspects of virtual rotations to include enhanced ease of interaction with faculty, a well-structured program and curriculum, student-focused case discussions, improved ability to identify critical patients, and improved familiarity and ability to work in a virtual healthcare setting [3,11]. Preceptors found the rotations to be innovative, progressive, and necessary [3].

Villa et al. reported matching three of their interns from their virtual clerkship [4]. Yellin et al. reported that 26.6% of candidates chosen to interview (17 out of 64) had taken part in their virtual rotation, and of those 17 candidates, eight were ranked within a “matchable range”, and of those applications, six were successfully matched into that residency program [10].

Disadvantages

Unfortunate disadvantages with this format included the lack of assessment of practical skills, especially in surgical rotations, lack of independent access to the EMR, sometimes students were not able to receive required school credit, and were not able to obtain a letter of recommendation [11,17,26]. With COVID-19 limiting the number of surgeries performed, students were not exposed to the entire range of those specialties [12].

In rotations that did not allow direct patient contact, there were decreased opportunities to practice history-taking, formulation of differential diagnoses, case presentations, and development of management plans. However, those rotations attempted to substitute patient contact with role-playing activities [5,12].

Other problems were experienced that were not unique to the field of medical education, including common technical problems such as poor internet connectivity, distractions, feelings of isolation, limited peer engagement, and difficulty fostering participation in an online learning environment [5,17].

The Future Outlook for Virtual Rotations

Medical students agreed that virtual rotations should be continued in the future based on the responses given in the studies by Villa et al. (95% of 25 respondents) [4] and Mason and Aruma (22 out of 27 respondents) [11].

It has been suggested that virtual rotations have aided the education and recruitment of students from various backgrounds and that they can be modified to occur, even when the COVID-19 spikes have resolved [3,14]. Recommendations for future rotations include increased involvement in virtual consultations with patients, recording didactic sessions for future participants to view, virtual seminars and simulations, streaming of procedures, exploring various combinations of rotations durations and formats, and increased application of evidence-based medicine [17,20].

It can thus be said that virtual rotations provided a good avenue for medical students to gain clinical exposure and training, at a time when their physical presence in the clinical spaces was not possible. Given the widespread development and acceptance of virtual rotations, it is reasonable to postulate that these rotations may continue well into the future.

Strengths and Limitations of the Study

This review evaluated concisely the existing published literature, exploring the widespread use of virtual rotations in various specialties and the rotations’ advantages and disadvantages. However, limitations were unavoidable and included the relative novelty of virtual rotations leading to a small number of papers to evaluate and the existence of virtual rotations which were not documented in published literature and hence were not recorded in this review. Another limitation is that more databases, and hence more studies, were not included.

## Conclusions

The COVID-19 pandemic has encouraged the implementation of “virtual rotations” which are a form of remote learning that seeks to emulate the clinical learning environment. We sought to explore which specialties adapted this format, the advantages, disadvantages, roles of the participants, and how well these rotations were able to substitute for in-person rotations. Through our database search, we included 23 relevant published articles for review. Virtual rotations have been developed in almost every specialty, throughout the United States, and in other countries. These rotations are targeted toward medical students, medical residents, and physician assistants and range in length from one to four weeks. While most rotations allowed case discussions, and didactic teaching sessions, some rotations allowed history-taking from patients, attending clinics, and visual diagnoses. Surgical specialties benefited from allowing students to live-stream surgical procedures and operations. Advantages included monetary savings, less time-consuming, increased numbers of participants, flexibility, and diversity. Virtual rotations participants have obtained interviews and matched to residency programs. Disadvantages included lack of assessment of practical skills, lack of independent access to the EMR, lack of school credit, inability to obtain letters of recommendation, decreased direct patient contact, and technical problems. Medical students agreed that virtual rotations should be continued. There have been suggestions for the implementation of virtual rotations even outside of the COVID-19 pandemic and thus have been a good substitute for in-person rotations.
